# In Situ Hepatitis C NS3 Protein Detection Is Associated with High Grade Features in Hepatitis C-Associated B-Cell Non-Hodgkin Lymphomas

**DOI:** 10.1371/journal.pone.0156384

**Published:** 2016-06-03

**Authors:** Danielle Canioni, Jean-Marie Michot, Pascaline Rabiega, Thierry J. Molina, Frédéric Charlotte, Thierry Lazure, Frédéric Davi, Catherine Settegrana, Françoise Berger, Laurent Alric, Patrice Cacoub, Benjamin Terrier, Felipe Suarez, David Sibon, Jehan Dupuis, Cyrille Feray, Hervé Tilly, Stanislas Pol, Bénédicte Deau Fischer, Sandrine Roulland, Catherine Thieblemont, Véronique Leblond, Fabrice Carrat, Olivier Hermine, Caroline Besson, national ANRS HC13 LymphoC study

**Affiliations:** 1 Department of Pathology, Paris Descartes University, AP-HP, Sorbonne Paris Cité, Hôpital Necker, Paris, France; 2 Department of Hematology and Drug Development, Gustave Roussy Institute, Villejuif, F-94805, France; 3 Institut Pierre Louis d’Epidémiologie et de Santé Publique INSERM UMR S1136, Paris 6 Pierre et Marie Curie University, Paris, France; 4 Department of Pathology, HôpitalPitié-Salpétrière, Paris 6 Pierre et Marie Curie University, Paris, France; 5 Department of Pathology, Paris 11 Sud University, AP-HP, Hôpital Bicêtre, Le Kremlin-Bicêtre, France; 6 Department of Biological Hematology, Paris 6 Pierre et Marie Curie University, AP-HP, Hôpital Pitié-Salpétrière, Paris, France; 7 Department of Pathology, Hopital Lyon Sud, Hospices Civils de Lyon, Centre Hospitalier Lyon Sud, Lyon, France; 8 Department of Internal Medicine and Digestive Diseases, Toulouse 3 University, UMR 152 IRD, Hôpital Purpan, Toulouse, France; 9 Department of Internal Medicine, APHP, Hôpital Pitié-Salpétrière, Paris 6 Pierre et Marie Curie University, UMR 7211, INSERM, UMR S 959, CNRS, Paris, France; 10 Department of Adult Hematology, Paris 5 Descartes University, AP-HP, Hôpital Necker, Paris, France; 11 Imagine Institute, University Sorbonne Paris Cité, INSERM U 1163, CNRS ERL 8254, Paris, France; 12 Department of Lymphoid Malignancies and Clinical Hematology, Paris 12 Est Créteil University, AP-HP, Hôpital Henri-Mondor, Créteil, France; 13 Department of Hepatology, Nantes University, Hôpital de Nantes, Nantes, France; 14 Department of Hematology, Rouen University, Centre Henri Becquerel, Rouen, France; 15 Department of Hepatology, Paris 5 Descartes University, INSERM U-1016, AP-HP, Hôpital Cochin, Paris, France; 16 Department of Hematology, Paris Descartes University, AP-HP, Hôpital Cochin, Paris, France; 17 Centre d'Immunologie de Marseille Luminy, Aix-Marseille Université INSERM U1104 CNRS UMR7280, Marseille, France; 18 Department of Hemato-oncology, University Paris Sorbonne P7, INSERM U728, AP-HP, Hôpital Saint-Louis, Paris, France; 19 Department of Clinical Hematology, Paris 6 Pierre et Marie Curie, Paris, France; 20 Department of Internal Medicine & Clinical Immunology Biological Immunology and Hematology, Paris 11 Sud University, AP-HP, Hôpital Bicêtre, INSERM U 1184, Le Kremlin-Bicêtre, France; University of Cincinnati College of Medicine, UNITED STATES

## Abstract

Hepatitis C Virus (HCV) infection is associated with the B-cell non-Hodgkin lymphomas (NHL), preferentially marginal zone lymphomas (MZL) and diffuse large B-cell lymphomas (DLBCL). While chronic antigenic stimulation is a main determinant of lymphomagenesis in marginal zone lymphomas (MZL), a putative role of HCV infection of B-cells is supported by in vitro studies. We performed a pathological study within the "ANRS HC-13 LymphoC" observational study focusing on in situ expression of the oncogenic HCV non structural 3 (NS3) protein. Lympho-C study enrolled 116 HCV-positive patients with B-NHL of which 86 histological samples were collected for centralized review. Main histological subtypes were DLBCL (36%) and MZL (34%). Almost half of DLBCL (12/26) were transformed from underlying small B-cell lymphomas. NS3 immunostaining was found positive in 17 of 37 tested samples (46%). There was a striking association between NS3 detection and presence of high grade lymphoma features: 12 out of 14 DLBCL were NS3+ compared to only 4 out of 14 MZL (p = 0.006). Moreover, 2 among the 4 NS3+ MZL were enriched in large cells. Remarkably, this study supports a new mechanism of transformation with a direct oncogenic role of HCV proteins in the occurrence of high-grade B lymphomas.

## Introduction

Hepatitis C Viral (HCV) infection is associated with B-cell non-Hodgkin lymphomas (NHL), preferentially marginal zone lymphomas (MZL) and diffuse large B-cell lymphomas (DLBCL) [1–5]. These NHL subtypes have different clinico-biological features and may differ with respect to mechanisms of lymphomagenesis. In most cases, HCV-related lymphomagenesis is consistent with a step wise model of transformation induced by chronic antigenic stimulation. Binding of the HCV envelope glycoprotein E2 to CD81 and to the B-cell receptor (BCR) could result in chronic stimulation of marginal B-cells. The stimulated B-cells produce rheumatoid factors (RF) leading to mixed cryoglobulinemia (MC). Chronic stimulation is thought to result in an accumulation of genetic lesions promoting the occurrence of overt MZL and/or eventually transforming in DLBCL [6].

An alternative mechanism is based on a direct transformation of B-cells following their infection by HCV. Indeed, although hepatocyte compartment is the main reservoir and replication site of the virus, HCV is also able to infect B-lymphocytes. Its replication in these cells could lead to oncogenic events mediated by the presence of intracellular viral proteins [6]. In vitro, expressions of HCV core protein (C) and non-structural protein 3 (NS3) have been found to induce nitricoxide synthase (NOS) and reactive oxygen species (ROS) generation, which might be responsible for mutations and abnormalities in DNA repair system and ultimately leading to cell transformation [7]. In vivo, the existence of extra-hepatic reservoirs is supported by the detection of viral RNA in B-cells of infected individuals [8]. The impact of the infection of B-cells on lymphomagenesis in vivo is largely unknown since direct evidence of viral proteins expression in lymphoma cells are scarce [9–10]. To better understand the role of HCV in lymphomagenesis in B-cell NHL, we performed a pathological study within the ANRS HC-13 LymphoC study [3], focusing on in situ the expression of the HCV NS3 protein. This study highlights the association between NS3 expression and the high grade NHL features.

## Materials & Methods

### Subjects

Adult patients with HCV-associated B-NHL were included in the observational multicentric ANRS HC-13 LymphoC study [3]. Investigations were performed after approval by a local Human Investigations Committee (Comité Consultatif pour la Protection des Personnes dans les Recherches Biomédicales [CCPPRB]) and approved by the French Department of Health and Human Services. Informed written consent was obtained from each subject. The study was registered in clinicalTrials.gov (Identifier number NCT01545544). Patients co-infected with HIV were excluded.

### Methods

Pathological specimens were collected for revision by a hematopathologists reviewing group (DC, TM, FC, TL, FB). Among the 116 patients enrolled in the main study, 86 cases were reviewed and included in the present histological study. Diagnoses of B-NHL were established on the basis of histological and immunophenotyping analysis and revised according to the WHO 2008 classification of lymphomas [11]. Immunophenotyping of circulating and/or medullar cells were performed in each center and revised by expert haematologists (CS, FD). In cases of discrepancy between the local center and the reviewing group, a multidisciplinary approach with clinician hematologists and hematopathologists was undertaken to determine B-NHL subtypes. A panel of antibodies was used for immuno-histochemistry studies following a classical three-step immunoperoxydase method on each fixed sample in order to classify B-cell NHLs (Table A in S1 File). DLBCL were subclassified according to the Hans algorithm whenever there was enough or appropriate material [12]. Presence of histological and immunological low-grade lymphoma features in DLBCL was classified as transformed-DLBCL; otherwise DLBCL were classified as de novo DLBCL.

Immunostaining with HCV NS3 antibody (Novocastra Lab) was performed in order to detect the in situ expression of HCV in 37 cases with enough or adequate material. For the other cases, either the block was too tiny for a valuable appreciation of the staining or the lymphoma on the specimen studied was necrotic and the staining not considered as valuable. Bone marrow biopsies were also excluded because of decalcification procedure which led to difficult interpretation. Paraffin sections from HCV-negative NHL (10 DLBCL and 5 MZL cases) were selected as negative controls. Paraffin sections from HCV-related chronic hepatitis (10 liver biopsies) were selected as positive controls.

### Statistical analysis

Descriptive statistics included the median and range for continuous variables and frequency (%) for categorical variables. Clinical and biological characteristics were compared between patients with positive and negative NS3 immunostaining. The Fisher’s exact tests was used to compare categorical variables, and the non-parametric Mann-Whitney test, to compare continuous variables. The duration between HCV infection and NHL was estimated from the time of occurrence of a potential risk factor for HCV infection (i.e. blood transfusion) to the time of NHL diagnosis. The differences were considered significant at p <0.05.

## Results

### Lymphoma characteristics

The characteristics of the ANRS HC 13 Lympho C patients were presented in the original study report [3]. Briefly, median age at B-NHL diagnosis was 61 years and gender ratio was 1. Among the 86 reviewed biopsies, the most frequent B-cell NHL subtypes were DLBCL (n = 31) and MZL (29). Sub-classification according to Hans algorithm could be performed in 26 DLBCLs. The majority (20/26) were of non-germinal center (GC) origin while 6 DLBCL were of GC subtype. Nine DLBCL were transformed from MZL and 3 from follicular lymphomas (FL). Lastly, there were 8 FL, 2 mantle cell lymphomas, 2 lymphocytic lymphomas and the remaining 14 cases were unclassified small B-cell lymphomas.

### HCV NS3 expression on lymphoma cells by immunostaining

**Fig 1:**
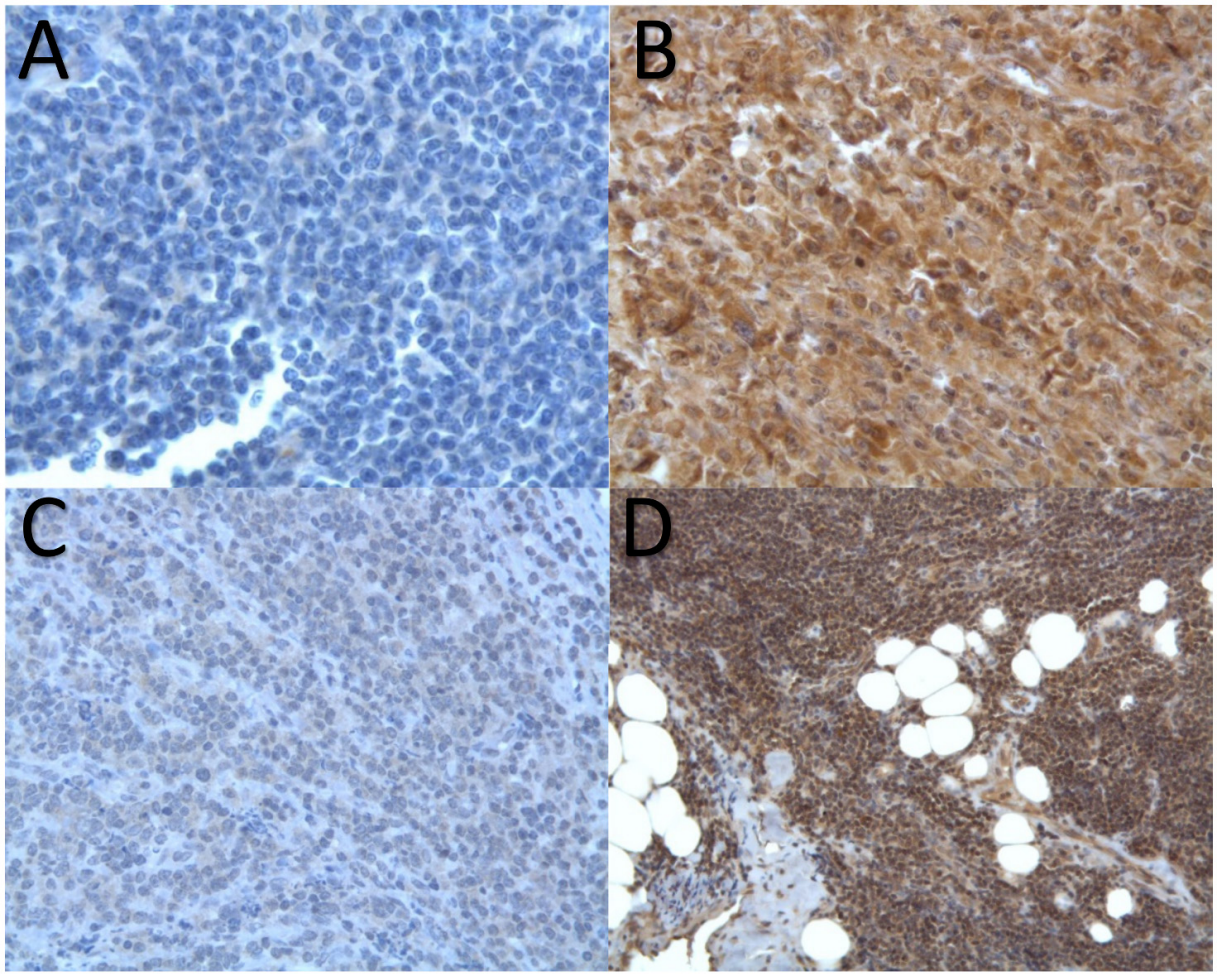
NS3 immunostaining in HCV associated non-Hodgkin’s lymphomas. A: Negative staining of a marginal zone lymphoma (MZL). B: Strong staining of a Diffuse Large B-Cell Lymphoma. C: Weak staining of a “classical” MZL. D: Strong staining of a MZL enriched in large cells.

**Fig 2:**
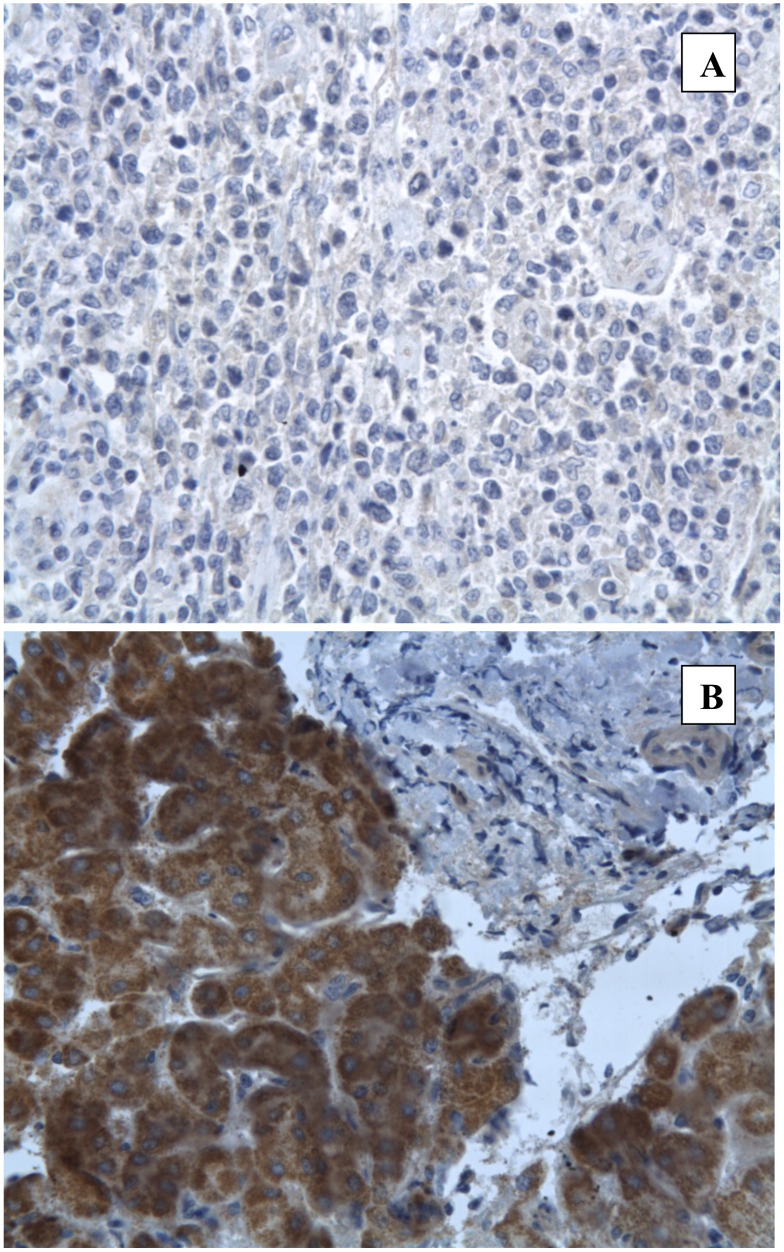
NS3 immunostaining in controls. A: Negative staining in a DLBCL biopsy from a HCV negative patient. B: Strong positive staining in a liver biopsy from a HCV positive patient without lymphoma.

NS3 expression could be detected in the cytoplasm of lymphoma cells with three patterns of staining either strongly positive, weakly positive or negative (Fig 1). Among the 37 NHLs that could be tested for NS3 expression by immunostaining, 17 cases were positive (Table 1). Remarkably, the proportion of NS3 positivity was strikingly higher among DLBCL than among MZL (86% vs 29%, p = 0.006)(Table 2). Twelve DLBCL were NS3 positive out of 14 tested. Of note, the five transformed DLBCL were NS3 positive. In contrast to DLBCL, only 4 out of 14 MZLs were NS3 positive. Remarkably, two of them were enriched in large cells (Table 1). Conversely, none of the 10 NS3 negative MZL were enriched in large cells. Among the nine cases of other subtypes of small B-cell NHL tested for NS3 expression, only one FL case was found positive. Finally, the 10 DLBCL and the 5 MZL biopsies from HCV negative patients, used as negative controls, showed no staining with NS3 whereas the liver biopsies of 10 HCV positive patients, used as positive controls, revealed a diffuse cytoplasmic staining (Fig 2).

**Table 1:** Demographic, histological characteristics and anti-NS3 staining in patients with HCV associated non-Hodgkin’s lymphomas (n = 37).

Patients	Sex	Age	Tumor site	Histology Immunohistology						
				Subtype	GC	NS3	bcl2	CD10	bcl6	MUM1
1	M	71	LNode	DLBCL	no	+	NT	-	NT	+
2	F	56	LNode	DLBCL	no	+	+	-	+	+
3	M	47	LNode	DLBCL	yes	+	+	+	+	+
4	M	60	Tonsil	DLBCL	no	+	+	-	+	-
5	F	81	LNode	DLBCL	no	+	+	-	NT	+
6	F	73	Salivary Gld	DLBCL	no	+	-	-	-	+
7	F	75	Skin	DLBCL	no	+	+	-	+	+
8	F	81	LNode	DLBCL	no	+	+	-	+	+
9	M	36	Liver	DLBCL	yes	+	+	+	+	+
10	M	49	Skin	DLBCL	yes	+	-	+	+	-
11	M	73	LNode	DLBCL	no	+	-	-	+	+
12	M	84	LNode	DLBCL	no	+	+	-	-	+
13	M	58	Liver	DLBCL	no	-	-	-	+	+
14	M	51	LNode	DLBCL	no	-	+	-	+	+
15	F	76	LNode	MZL LC	-	+	+	-	-	+
16	F	62	Orbit Muscle	MZL LC	-	+	+	-	+	+
17	M	80	LNode	MZL	-	+	+	-	-	-
18	M	67	Lung	MZL	-	+	+	-	-	NT
19	M	63	LNode	MZL	-	-	+	-	-	NT
20	M	60	LNode	MZL	-	-	+	-	-	-
21	F	52	Spleen	MZL	-	-	+	-	-	-
22	F	82	Liver	MZL	-	-	+	-	-	-
23	F	79	Skin	MZL	-	-	+	-	-	+
24	M	42	Stomach	MZL	-	-	+	-	NT	NT
25	F	61	Eye	MZL	-	-	+	-	NT	-
26	F	59	LNode	MZL	-	-	+	-	NT	+
27	M	87	Liver	MZL	-	-	+	-	NT	+
28	F	77	Skin	MZL	-	-	+	NT	-	
29	F	71	LNode	FL	-	+	+	+	+	NT
30	M	62	LNode	FL	-	-	+	+	+	NT
31	F	76	LNode	FL	-	-	+	+	-	-
32	F	65	LNode	FL	-	-	+	-	+	+
33	F	71	Duodenum	FL	-	-	+	+	+	-
34	F	61	LNode	FL	-	-	+	+	+	NT
35	M	55	LNode	MCL	-	-	+	-	NT	+
36	F	45	LNode	MCL	-	-	+	-	NT	+
37	F	75	LNode	CLL	-	-	+	-	NT	+

**Abbreviations**: CLL: chronic lymphocytic leukemia; DLCL: diffuse large B-cell lymphoma; FL: follicular lymphoma; GC: germinal center; Gld: gland; LC: enriched in large cells; LNode: lymph node; MCL: mantle cell lymphoma; NT: not tested.

**Table 2:** Comparison of patients’ characteristics according to NS3 immunostaining.

	NS3 negative (n = 20)	NS3 positive (n = 17)	p-value
**Male / Female ratio**	8 / 12	9 / 8	0.62
**Age (years)** Median [range]	61 [42–87]	67 [36–84]	0.97
**Lymphoma type**			0.006 ^b^
DLBCL	2	12	
MZL	10	4	
Others ^a^	8	1	
**LDH**			0.07
Normal	12	7	
Above normal value	3	9	
Unknown	5	1	
**HCV viral load at lymphoma diagnosis** Median [range], Log(copies/ml)	6.1 [1.1–7.2]	6.5 [0–7.7]	0.82
**HCV Genotype**			0.50^c^
**1**	12	7	
**2**	4	2	
**Others**	3	5	
**Unknown**	1	3	

**Abbreviations:** DLBCL: diffuse large B-cell lymphoma; FL: follicular lymphoma, MZL: Marginal Zone lymphomas.

Legend:

^a^: Others are 6 FL, 2 mantle cell lymphomas and one chronic lymphocytic leukemia

^b^: Comparison between DLBCL and MZL

^c^: Comparison between genotype 1 and others

### Patients’ characteristics according to NS3 immunostaining

There was no significant difference with respect to demographic features and lymphoma sites, between NS3 negative and positive cases. Patients with NS3 positive NHL tend to have higher serum LDH level (p = 0.07) than those with NS3 negative NHL (Table 2). Viral load at NHL diagnosis, delay between infection and lymphoma diagnosis, and virus genotypes did not differ between NS3 positive and negative cases (Table 2). Remarkably, one patient (patient #2, Table 1) had a NS3 positive DLBCL and a negative plasmatic HCV viral load at NHL diagnosis. This 56 year-old female patient had received a successful anti-viral therapy with pegylated interferon plus ribavirin the year before NHL diagnosis. Although she had achieved a sustained virological response, she developed a DLBCL with a positive NS3 staining nine months after the end of antiviral treatment. Her HCV viral load remained negative during the lymphoma treatment and follow-up.

## Discussion

In this histological study, we confirm the predominance of DLBCL and MZL in HCV-associated B-NHL as previously reported [3]. Pathological review underlined a continuum from low-grade to aggressive lymphomas, illustrated in our cohort by a third of DLBCL originating from MZL transformation. Strikingly, we found a strong correlation (p = 0.006) between NS3 *in situ* expression and DLBCL. Emphasizing this finding, two of the four NS3 positive MZL cases were enriched in large cells suggesting that in situ HCV infection of lymphoma B-cells could constitute a major event in their transformation in large cells. Our results are in line with the recent identification of a biological role of NS3 in BCR signaling [13]. This study demonstrated the expression of HCV NS3/4a viral proteins in primary B-cells from patients infected with HCV and highlighted their impact in the upregulation of BCR signaling. Altogether, these in vitro findings and the present in situ results support a novel molecular mechanism underlying HCV-associated B-cell lymphoma.

Few reports had previously revealed in situ detection of HCV proteins on NHL tissues [9–10]. However, the number of NHL tested cases in those studies was too limited to draw firm conclusions on their associations with NHL subtypes or characteristics. Our data showing the presence of NS3 in 12 out of 14 cases of DLBCL support that transformation of B-cells towards DLBCLs is favoured by their infection by HCV. In contrast, the negativity of NS3 staining in most of MZL suggests that lymphomagenesis in these cases may rely on other mechanisms such as chronic antigenic stimulation. These two mechanisms of transformation are not exclusive since NS3 positive staining was found in transformed DLBCL from MZL as well, suggesting that secondary infection of chronically stimulated MZL may participate to transformation.

In HCV associated MZL, the clearance of viral load following antiviral treatment is frequently associated with the control of lymphoma [14], suggesting that these malignant B-cells remain addicted to the BCR signalling induction by the virus. Our finding of an expression of the viral NS3 protein within the cells of aggressive B- NHL supports a possible benefice of viral eradication also in this setting. Altogether, according to our data as well as the literature showing an efficacy of immunochemotherapy in HCV related DLBCL and a positive impact of antiviral therapy on survival of patients with HCV related lymphomas [3], we infer that DLBCL require immunochemotherapy associated with antiviral therapy either in combination or sequentially. Finally, the medical history of patient #2 who developed a NS3 positive DLBCL after having successfully received anti-viral therapy, supports that HCV infection may persist in human B-cells compartment in patients in sustained virological response.

A limit of the study is that we did not have enough material to perform RT-PCR analysis to assess mRNA virus expression in lymphoma cells to validate protein expression detected by immunohistochemistry. However, in two previous studies mentioned above, NS3 staining combined with RT-PCR showed that HCV RNA could be detected in all the NS3 positive cases [10,15], validating immunohistochemistry analysis as a surrogate marker of B-cell infection. In conclusion, this study highlights the heterogeneity of B-NHL phenotypes associated with HCV, with a real continuum between MZL, MZL enriched in large cells and transformed DLBCL. In this transformation process, the viral protein NS3 appears to be associated with the DLBCL subtype. Therefore, in addition to the role of chronic antigenic stimulation in HCV related lymphomagenesis, this study supports a second mechanism of transformation due to a direct oncogenic role of HCV infection of B-cells promoting the occurrence of high-grade B-cell lymphomas.

## Supporting Information

S1 File Table A. Characteristics of the antibodies used in immunohistochemistry. Table B. Supplementary data set of the study patients with HCV associated non Hodgkin’s lymphomas (n = 37) and NS3 immunostaining.
(DOCX)
