# Supplementary material for: In Situ Hepatitis C NS3 Protein Detection Is Associated with High Grade Features in Hepatitis C-Associated B-Cell Non-Hodgkin Lymphomas
Source: PLoS One. 2016 Jun 3;11(6):e0156384. doi: 10.1371/journal.pone.0156384 (PMC4892517; doi:10.1371/journal.pone.0156384)
Supplement: S1 File — Table A. Characteristics of the antibodies used in immunohistochemistry. Table B. Supplementary data set of the study patients with HCV associated non Hodgkin’s lymphomas (n = 37) and NS3 immunostaining. (DOCX) [file pone.0156384.s001.docx]

**Supporting Information**

**Table A. Characteristics of the antibodies used in immunohistochemistry**

|  | **Lab** | **Clone** | **Dilution** | **City** | **Country** |
| --- | --- | --- | --- | --- | --- |
| **CD20** | Dako | L26 | 1/400 | Glostrup | Denmark |
| **CD3** | Dako | Polyclonal | 1/200 | Glostrup | Denmark |
| **CD5** | Novocastra | 4C7 | 1/100 | Newcastle | Great Britain |
| **bcl2** | Dako | 124 | 1/200 | Glostrup | Denmark |
| **bcl6** | Dako | PG-B6p | 1/25 | Glostrup | Denmark |
| **CD10** | Novcastra | 270 | 1/25 | Newcastle | Great Brttain |
| **MUM1** | Dako | MUM-1p | 1/50 | GLostrup | Denmark |
| **CD23** | Novocastra | 1B12 | 1/50 | Newcastle | Great Britain |
| **CD21** | Novocastra | 2G9 | 1/15 | Newcastle | Great Britain |
| **CD138** | Dako | MI15 | 1/50 | Glostrup | Denmark |
| **Cyclin D1** | ThermoScientific | SPA | 1/20 | Fremont | USA |
| **KI67** | Dako | MIB-1 | 1/100 | Glostrup | Denmark |
| **Kappa** | Diagomics | Kap56 | 1/100 | Santa Barbara | USA |
| **Lambda** | Diagomics | Lamb14 | 1/100 | Santa Barbara | USA |
| **IgA** | Dako | Polyclonal | 1/2000 | Glostrup | Denmark |
| **IgG** | Dako | Polyclonal | 1/10000 | Glostrup | Denmark |
| **IgM** | Dako | Polyclonal | 1/10000 | Glostrup | Denmark |
| **NS3** | Novocastra | MMM33 | 1/25 | Newcastle | Great Britain |

**Table B. Supplementary data set of the study patients with HCV associated non Hodgkin’s lymphomas (n=37) and NS3 immunostaining**

| **Patients** | **Sex** | **Age** | | **Tumor site** | | **Histology** | **NS3** | | | **HCV VL**  **(Log EQ/ml)** | |  | **LDH** | | |
| --- | --- | --- | --- | --- | --- | --- | --- | --- | --- | --- | --- | --- | --- | --- | --- |
| 1 | M | 71 | LNode | | DLBCL | | | + | 6.2 | | | >UNL | | |  |
| 2 | F | 56 | LNode | | DLBCL | | | + | 0.0 | | | >UNL | | |  |
| 3 | M | 47 | LNode | | DLBCL | | | + | 6.5 | | | >UNL | | |  |
| 4 | M | 60 | Tonsil | | DLBCL | | | + | 5.9 | | | N | | |  |
| 5 | F | 81 | LNode | | DLBCL | | | + | 4.7 | | | N | | |  |
| 6 | F | 73 | Salivary Gld | | DLBCL | | | + | 7.1 | | | >UNL | | |  |
| 7 | F | 75 | Skin | | DLBCL | | | + | 6.9 | | | >UNL | | |  |
| 8 | F | 81 | LNode | | DLBCL | | | + | 7.7 | | | >UNL | | |  |
| 9 | M | 36 | Liver | | DLBCL | | | + | 6.0 | | | N | | |  |
| 10 | M | 49 | Skin | | DLBCL | | | + | 5.8 | | | N | | |  |
| 11 | M | 73 | LNode | | DLBCL | | | + | 5.4 | | | N | | |  |
| 12 | M | 84 | LNode | | DLBCL | | | + | 5.6 | | | >UNL | | |  |
| 13 | M | 58 | Liver | | DLBCL | | | - | 5.1 | | | >UNL | | |  |
| 14 | M | 51 | LNode | | DLBCL | | | - | 1.1 | | | N | | |  |
| 15 | F | 76 | LNode | | MZL LC | | | + | 6.7 | | | >UNL | | |  |
| 16 | F | 62 | Orbit Muscle | | MZL LC | | | + | 6.5 | | | N | | |  |
| 17 | M | 80 | LNode | | MZL | | | + | 6.6 | | | N | | |  |
| 18 | M | 67 | Lung | | MZL | | | + | 6.5 | | | >UNL | | |  |
| 19 | M | 63 | LNode | | MZL | | | - | 6.5 | | | NA | | |  |
| 20 | M | 60 | LNode | | MZL | | | - | 5.7 | | | N | | |  |
| 21 | F | 52 | Spleen | | MZL | | | - | 4.7 | | | N | | |  |
| 22 | F | 82 | Liver | | MZL | | | - | 7.1 | | | NA | | |  |
| 23 | F | 79 | Skin | | MZL | | | - | 6.5 | | | NA | | |  |
| 24 | M | 42 | Stomach | | MZL | | | - | 5.8 | | N | | |  |  |
| 25 | F | 61 | Eye | | MZL | | | - | 4.9 | | N | | |  |  |
| 26 | F | 59 | LNode | | MZL | | | - | 6.0 | | >UNL | | |  |  |
| 27 | M | 87 | Liver | | MZL | | | - | 6.1 | | N | | |  |  |
| 28 | F | 77 | Skin | | MZL | | | - | NA | | N | | |  |  |
| 29 | F | 71 | LNode | | FL | | | + | 6.7 | | NA | | |  |  |
| 30 | M | 62 | LNode | | FL | | | - | NA | | NA | | |  |  |
| 31 | F | 76 | LNode | | FL | | | - | 6.2 | | N | | |  |  |
| 32 | F | 65 | LNode | | FL | | | - | 5.9 | | N | | |  |  |
| 33 | F | 71 | Duodenum | | FL | | | - | 6.3 | | NA | | |  |  |
| 34 | F | 61 | LNode | | FL | | | - | 6.0 | | N | | |  |  |
| 35 | M | 55 | LNode | | MCL | | | - | 6.7 | | N | | |  |  |
| 36 | F | 45 | LNode | | MCL | | | - | 7.2 | | >UNL | | |  |  |
| 37 | F | 75 | LNode | | CLL | | | - | 6.8 | | N | | |  |  |

**Abbreviations**: CLL: chronic lymphocytic leukemia; DLCL: diffuse large B-cell lymphoma; FL: follicular lymphoma; MCL: mantle cell lymphoma; N: within normal range; NA: not available; UNL: upper normal limit; VL: viral load.
